# Safety and Efficacy of Erythropoietin in Traumatic Brain Injury Patients: A Pilot Randomized Trial

**DOI:** 10.1155/2010/209848

**Published:** 2010-05-12

**Authors:** R. Nirula, R. Diaz-Arrastia, K. Brasel, J. A. Weigelt, K. Waxman

**Affiliations:** ^1^Division of General Surgery, University of Utah School of Medicine, 3B148, 30 North 1900 East, Salt Lake City, UT 84132, USA; ^2^Division of Neurology, UT Southwestern Medical Center, Dallas, TX 75390, USA; ^3^Division of Trauma and Critical Care, Medical College of Wisconsin, Milwaukee, WI 53226, USA; ^4^Department of Surgery, Santa Barbara Cottage Hospital, Santa Barbara, CA 93105, USA

## Abstract

*Background*. Erythropoietin (EPO) is a neuroprotective agent utilized in stroke patients. This pilot study represents the first randomized trial of EPO in traumatic brain injury (TBI) patients. *Methods*. Adult, blunt trauma patients with evidence of TBI were randomized to EPO or placebo within 6 hours of injury. Baseline and daily serum S-100B and Neuron Specific Enolase (NSE) levels were measured. *Results*. TBI was worse in the EPO (*n* = 11) group compared to placebo patients (*n* = 5). The use of EPO did not impact NSE (*P* = .89) or S100 B (*P* = .53) levels compared to placebo. *Conclusions*. At the dose used, EPO did not reduce neuronal cell death compared to placebo; however, TBI severity was worse in the EPO group while levels of NSE and S100-B were similar to the less injured placebo group making it difficult to rule out a treatment effect. A larger, balanced study is necessary to confirm a potential treatment effect.

## 1. Introduction

Despite injury prevention efforts, severe head injury is estimated to occur at a rate of 200 per 100,000 people in the United States [1]. Close to 100,000 head injured patients suffer permanent disability or death each year. The economic consequences of this are equally impressive with 30 million workdays lost and a societal cost of 83.5 billion dollars per year [1, 2]. 

Traumatic brain injury follows a well-recognized pattern beginning with the primary injury due to the traumatic event. The neuronal loss from this event is irrecoverable; however, regions surrounding these damaged sites are subject to alterations in cerebral blood flow regulation, edema, inflammation, apoptosis, and ischemia. Furthermore, these regions may have increased metabolic demands, thereby increasing the disparity between oxygen requirements and supply. These vulnerable areas, known as the ischemic penumbra, reflect regions of the brain prone to secondary brain injury [3, 4]. 

As a result, current management principles of the head injured focus upon prevention of secondary brain injury in order to maximize the neurologic outcome. The strategies employed reflect the need to provide well-oxygenated blood flow to these vulnerable regions of the brain [1, 4–7]. These strategies, however, do not primarily manipulate neuronal cellular activity and substrate requirements, but instead deal only with maintenance of adequate substrate supply. 

Recently, several investigations have demonstrated that EPO has time-dependent neuroprotective effects in animal models independent of its role in erythropoiesis [8–14]. The mechanisms by which EPO exerts its effects on neuronal cells are not clearly understood, but evidence suggests that it mediates cerebral vasoconstriction, glutamate toxicity, neuronal antioxidant activity, endothelial cell apoptosis, and nitric oxide release [8–15]. Several lines of evidence indicate that EPO, administered peripherally, may cross the blood brain barrier through a specific transport mechanism which may be up-regulated during cerebral hypoxia [16–18]. Taken together, these studies suggest a therapeutic role for EPO in head injured patients during their resuscitation. The safety and efficacy of EPO in TBI patients has yet to be assessed clinically. We, therefore, proposed a randomized, double-blinded, placebo-controlled, pilot study to determine if EPO, given to moderate traumatic brain injury (TBI) patients during the resuscitative phase, would be safe and could lead to a measurable reduction in neuronal cell death as measured by neuron-specific cell markers.

## 2. Methods

This was a randomized, double-blind, placebo-controlled single-center trial in which patients either received EPO or saline placebo of equal volume. All blunt trauma patients ≥18 years of age with an admission GCS < 13 and evidence of traumatic brain injury (TBI) on CT were eligible for this IRB approved study. After obtaining informed consent from the family, patients were randomized to receive EPO (40,000 Units IV) or placebo administered within 6 hours of the time of injury. The primary outcome measure was S-100B and NSE levels in patients receiving EPO were compared to those receiving placebo. The sample size estimate was based upon an anticipated reduction of 25% in S-100B levels with EPO treatment compared to placebo. The sample size required to detect such a difference with type II error of 0.9 and type I error of 0.05, using a two-sided binomial hypothesis, was 43 patients per group. Assuming a 25% drop out rate for early nontraumatic brain injury-related mortality and/or refused consent from family members after enrollment, the planned enrollment was 108 patients (54 per group) into the study. The study only enrolled 23 patients due to the fact that the primary investigator had moved to another institution prior to study completion. 

Patients had baseline (day of injury) and daily serum S-100B and NSE levels measured until 5 days after injury. Demographic and clinical data were obtained including age, gender, head AIS, ISS, admission and ICU GCS, daily mean ICP and CPP (when ICP was monitored), number and nature of ICP lowering interventions, and daily mean PaCO2. Secondary outcome measures included ICU LOS, GCS at ICU discharge, and in-hospital mortality. CT scans were independently reviewed by a radiologist (blinded to the randomization) using a standardized technique for quantifying head injury severity that correlates with outcome [19]. 

Blood samples were collected by venipuncture upon admission and approximately every 24 hours thereafter until 5 days post injury. Samples were allowed to clot for 20–30 minutes at room temperature and centrifuged at 800–1000 RPM for 10 minutes and stored at −18°C for analysis. S-100B and NSE were analyzed in duplicate using a monoclonal 2-site immunoluminometric assay kit (Santeg 100 and NSE prolifigen; AB Santeg Medical). Sample and luminescence-labeled anti-S-100B or anti-NSE were added to the antibody-coated tube and then incubated. Unbound material was removed by washing. Tracer-S-100B or NSE complex bound to the tube wall was detected by light reaction (425 nm) produced by the reaction as measured using a liminometer. The light signal, measured in relative light units, was therefore directly proportional to the amount of NSE or S-100 present in the standard and sample. 

Baseline patient characteristics were compared using Student *t*-test for continuous variables and chi-square testing for categorical variables. A multivariate repeated measures analysis of covariance (ANCOVA) was performed to ascertain the effect of EPO on NSE and S-100B serum levels between groups over time while adjusting for differences in head injury severity. For all analyses, *α* was set at 0.05. Analyses were conducted using Stata Statistical Software (release 9.0, Stata Corp, College Station, Tex).

## 3. Results

**Figure 1:**
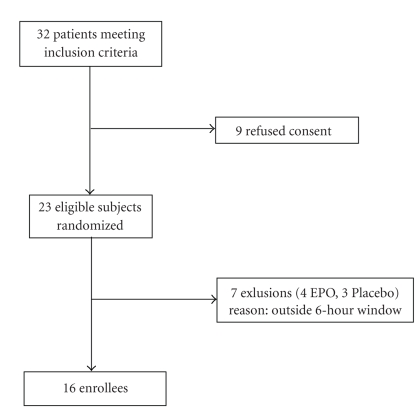
Study population.

A total of 23 patients were initially randomized with 7 patients dropping out before receiving the study agent leaving 16 patients (EPO *n* = 11, placebo *n* = 5) for subsequent analysis. The 7 dropouts were patients who were initially randomized but never received the study agent or placebo because they were found to be outside of the six-hour inclusion criteria by the time the agent was available (Figure 1).

The groups were similar in terms of demographics, and overall injury severity; however, CT rating of the head injury severity showed that the EPO group had significantly more severe injury at baseline despite randomization (Table 1). The EPO group was also found to have a lower admission GCS compared to the placebo group (5.4 versus 8.3, resp., *P* = .1) despite the fact that the head abbreviated injury score was similar for both groups. 

**Figure 2:**
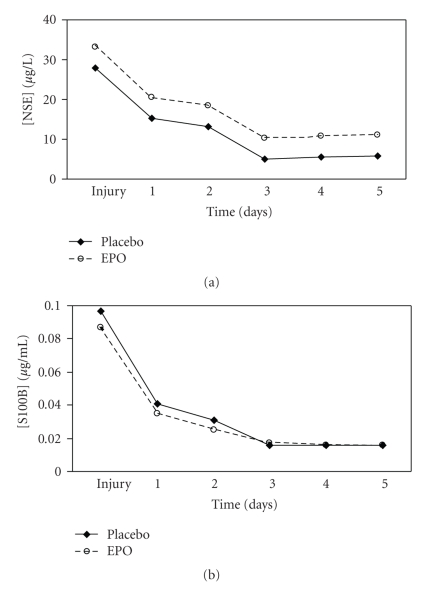
(a) NSE Serum levels in patients receiving EPO or Placebo. (b) S-100B Serum levels in patients receiving EPO or Placebo.

**Figure 3:**
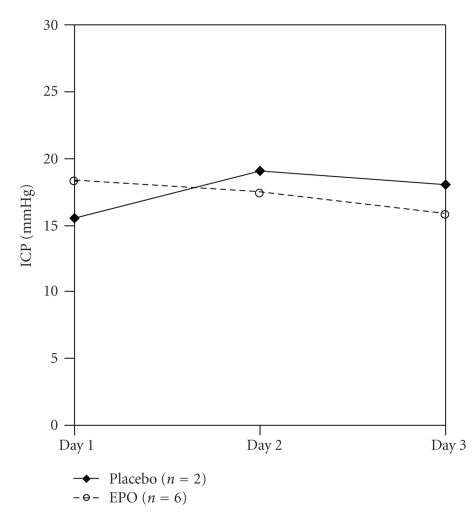
Mean daily ICP by study group.

Daily mean S-100B and NSE levels were initially elevated and then declined significantly (*P* < .0001) in both groups over the first three days but remained detectable throughout the 5-day measurement period (Figures 2(a) and 2(b), Table 2). The use of EPO did not significantly impact NSE (*P* = .89) or S-100B (*P* = .53) levels compared to placebo over the 5 day period despite adjustment for head injury severity. Mean daily ICP values were not different between the two groups (Figure 3). There were two in-hospital deaths in the EPO group and none in the placebo group (*P* = NS). One patient died from his head injury and the other died from hypoxia from ARDS. One episode of DVT occurred in the placebo group and none in the EPO group (*P* = NS). Mean maximum ICP levels were similar for each of the 5 days between the two groups. ICU length of stay, was shorter for the EPO group by a mean of 2 days, with and without the two deaths, but this did not reach statistical significance.

## 4. Discussion

This preliminary analysis did not demonstrate a reduction in neuronal cell death as determined by serum markers when EPO was administered at a dose of 40,000 units within 6 hours of injury. Secondary outcomes of death, length of stay and Glascow outcome scores also did not differ with the treatment. 

Several limitations in this study affect our ability to draw any strong conclusions from the absence of an effect. First, the study is underpowered for its primary outcome of NSE and S-100B levels as a result of premature study cessation secondary to reasons not associated with the study itself. As a result, a lack of treatment effect may not be secondary to lack of efficacy but because of inadequate sample size. Second, while NSE and S-100B have been correlated with the presence and severity of head injury, they may not be sufficiently sensitive to correlate with any beneficial neuroprotective effects that EPO may be having. Recently, several investigations have identified lipid membrane peroxidation as a major contributor to blood brain barrier breakdown and hence neuronal death [20–22]. Furthermore, these byproducts of lipid peroxidation are vasoreactive leading to cerebrovasoconstriction, further compromising neuronal cell longevity [22]. F2-isoprostaglandin is one such marker of lipid peroxidation and has been used to quantify the extent of traumatic brain injury and response to antioxidant therapies, such as progesterone. In fact, CSF levels of F2-isoprostane have been quantified as a useful marker of TBI in children demonstrating dramatic elevations compared to control groups [20, 23]. In adults the degree of oxidative stress appears to differ by gender and age after TBI as determined by differences in the levels of F2-ispoprostanes between males and females and older versus younger patients [24]. These data indicate that the isoprostanes are a useful marker of TBI severity and are modulated by therapies that reduce oxidative stress after TBI. Future studies should therefore make use of this biomarker of neuronal injury to quantify treatment effects. 

Several studies in the stroke literature using EPO as a neuroprotective agent have examined functional outcomes far beyond the short-term laboratory markers examined in this pilot study. The 5-day follow up in this trial was chosen based upon previous trials in the stroke and TBI literature which identified that NSE and S-100B levels reach baseline levels within 3–5 days of injury. 

As this is a preliminary study, the ideal dose to achieve a treatment effect is not yet known. In stroke patients higher doses of EPO, given over longer duration, have proven to be neuroprotective [25, 26]. Ehrenreich et al. studied the safety and efficacy of EPO in stroke patients using a dose of 33,000 IU given intravenously daily for three days. Their study demonstrated that CSF concentrations of EPO increased 60–100-fold compared to the placebo group and there was a correlation between EPO use and improved outcomes with smaller infarct size [26]. Subsequent studies in TBI patients will likely need to use similar doses to ensure adequate CSF penetration for a beneficial effect to be clearly observed. 

Any study of TBI which includes polytrauma patients may suffer from confounding secondary to the influence of other injuries on outcomes. In this study, we included patients with extracranial injuries which clearly has an impact on mortality and neurologic recovery. The severity of the extracranial injuries was not significantly different between the two groups which would reduce the degree of confounding. Furthermore, since the primary outcome of the study was early postinjury neurospecific proteins the effect of extracranial injuries would likely be minimal unless these injuries contributed significantly to the ability to provide adequate cerebral perfusion or significant hypoxia. As is shown in Table 1, there was no significant hypotension in either groups suggesting that the injuries were not likely a significant cause of early cerebral hypoxia. 

While this study failed to show efficacy, this should not deter further research regarding the use of EPO in TBI patients. Despite the randomization process, the small sample size leads to a disproportionately greater degree of head injury in the EPO group relative to the placebo group. In all of the radiographic measures of head injury severity assessed, the EPO patients were more severely injured. Again, the small sample size contributes to the lack of a statistical relationship being observed but one cannot overlook the fact that the radiographic appearance differed in terms of severity between the two groups in a clinically meaningful way. It is noteworthy that, in spite of this difference in head injury severity, EPO patients' NSE and S-100B levels fell as rapidly and to the same degree as the placebo group. It is therefore possible that, had these patients not received EPO, the degree of neuronal death observed may have been greater and may have been associated with higher NSE and S-100B levels than what we observed. This is purely speculation, however, and a larger trial with a dose-finding strategy is needed to determine if EPO is neuroprotective in TBI patients. This study demonstrates the safety of its use and the need for higher dosing in future studies.

## Figures and Tables

**Table 1:** Characteristics of patients receiving erythropoietin or placebo.

Characteristic (SD)	EPO	Placebo	*P*-value
	(*n* = 11)	(*n* = 5)	
Age	35 (19)	40 (26)	NS
Male	73%	60%	NS
Admit GCS	4.7 (3.3)	8.8 (5.3)	.04
Admit SBP	130 (33)	115 (26)	NS
Admit HR	91 (18)	78 (18)	NS
ICU GCS	11 (3.6)	9.8 (4.9)	NS
**Sedation on arrival**	**73%**	**40%**	**NS**
ISS	27 (8.4)	21.6 (8.4)	NS
Head AIS	4 (0.6)	3.8 (0.4)	NS
Midline shift (mm)	2.0 (3.4)	0 (0)	*P* = .12
EAH grade	6.0 (8.1)	0.3 (0.6)	*P* = .07
SDH grade	4.2 (7.3)	0 (0)	*P* = .12
EDH grade	1.8 (5.3)	0.3 (0.6)	NS
ICH grade	11.5 (12.7)	5.6 (9.0)	NS
SAH grade	3.2 (1.0)	3 (1.4)	NS

GCS: Glascow Coma Score, SBP: Systolic Blood Pressure, HR: Heart Rate, ICU GCS: first recorded GCS in the ICU, ISS: Injury Severity Score, AIS: Abbreviated Injury Severity Score, EAH: Extra-axial Hemorrhage, SDH: Subdural Hemorrhage, EDH: Epidural Hemorrhage, ICH: Intracranial Hemorrhage, SAH: Subarachnoid Hemorrhage.

**Table 2:** Daily NSE and S-100B levels by study group.

	NSE		S-100B	
Day	EPO	Placebo	EPO	Placebo
Admission	34.4 (20.9)	24.9 (21.0)	0.087 (0.04)	0.094 (0.05)
1	20.0 (9.9)	16.4 (6.4)	0.035 (0.01)	0.041 (0.03)
2	20.3 (16.4)	9.4 (4.0)	0.025 (0.009)	0.031 (0.02)
3	9.5 (10.5)	7.8 (7.6)	0.017 (0.006)	0.016 (0.004)
4	8.9 (7.3)	11.2 (13.4)	0.016 (0.005)	0.016 (0.004)
5	10.8 (9.2)	4.1 (1.8)	0.016 (0.005)	0.016 (0.003)
